# Predictors of unmet needs in Chilean older people with dementia: a cross-sectional study

**DOI:** 10.1186/s12877-019-1131-1

**Published:** 2019-04-15

**Authors:** Thamara Tapia Muñoz, Andrea Slachevsky, María O. León-Campos, Michel Madrid, Alejandra Caqueo-Urízar, Gustav C. Rohde, Claudia Miranda-Castillo

**Affiliations:** 1 Mailman School of Public Health, Columbia University, New York, USA; 2 Gerosciences Center for Brain Health and Metabolism (GERO), Santiago, Chile; 3 Neuropsychology and Clinical Neuroscience Laboratory (LANNEC), Physiopathology Department, Faculty of Medicine, Universidad de Chile, Santiago, Chile; 4 Physiopathology Department, ICBM, Neurosciences Department, East Neuroscience Department, Faculty of Medicine, Universidad de Chile, Santiago, Chile; 5 Memory and Neuropsychiatric Clinic (CMYN) Neurology Department, Hospital del Salvador & University of Chile, Santiago, Chile; 6 Servicio de Neurología, Departamento de Medicina, Clínica Alemana-Universidad del Desarrollo, Santiago, Chile; 7 Universidad Andres Bello, Faculty of Nursing, Sazié 2212, Santiago, Chile; 8 Millennium Institute for Research in Depression and Personality (MIDAP), Santiago, Chile; 9 Departamento de Filosofía y Psicología, Universidad de Tarapacá, Arica, Chile; 10 Facultad de Medicina, Universidad de Valparaíso, Valparaíso, Chile

**Keywords:** Needs, Dementia, Older people, Social support, Caregivers, Alzheimer’s disease, Mental health

## Abstract

**Background:**

The needs of people with dementia (PWD) have not been assessed in any Latin American country. Several European countries have already related unmet needs with quality of life, caregiver’s age, burden, stress, anxiety and depression. The aim of this study was to identify met and unmet needs in Chilean older adults with dementia and to determine if those needs were associated with PWD’s, their informal caregivers’ and social factors.

**Method:**

This was a cross-sectional study. One-hundred and sixty-six informal caregivers and their care recipients were interviewed. PWD was assessed about cognitive function and their caregivers answered instruments about PWD’s needs, functional status and behavioral and psychological symptoms. Caregiver’s burden, depression, anxiety and social support were also evaluated. A stepwise multiple linear regression analysis was performed to determine predictors of unmet needs in Chilean PWD.

**Results:**

The most frequent met needs were “Looking after home” (81.3%%), “Food” (78.9%) and “Selfcare” (75.3%). Most common unmet needs were “Daily living activities” (39.2%), “Company” (36.1%), and “Memory” (34.9%). Caregivers’ lower age was correlated to a higher number of PWD’s unmet needs (r_s_ = −.216; *p* < 0.005). Higher PWD’s dependence was associated with higher number of unmet needs (r_s_ = .177; *p* < 0.05). The best predictors of unmet needs were caregivers’ low level of social support, high burden, young age and high level of anxiety.

**Conclusion:**

It is necessary to address psychological and social needs of PWD. The fact that PWD’s unmet needs were associated mostly with caregivers’ factors, highlights the importance of considering both, the PWD and their informal caregivers as targets of institutional support. It is expected that recently launched national public policies decrease PWD’s unmet needs by the provision of new services for them and their informal caregivers.

## Background

Over 47 million of people have dementia worldwide [1]. Every year there is an approximate increase of 7.7 million cases at a global scale, which means a new case arises every 4 sec [2]. Dementia research has relied mostly in the biomedical model (etiology and treatment) without considering the psychological and social aspects of the disease [3].

In Chile, it has been estimated that 1.06% of the population over 60 years old (around 200,000 people) have some kind of dementia [4, 5]. Dementia care has been mostly a family responsibility, with isolated actions from the healthcare system such as a primary care screening for cognitive impairment and dementia [6, 7]. Chile is currently starting to pilot the first national strategy for dementia, which includes new initiatives to address this condition [8, 9]. In this context, in order to provide appropriate interventions and social services, the assessment of needs in PWD becomes crucial [6, 10, 11]. Needs can be studied considering the person’s own perspective (subjective need) or through the perception of a third one (objective needs). In the case of old adults with dementia, this external person usually is an informal caregiver or a health professional [12, 13].

The needs of PWD have already been studied in European countries. According with those studies, unmet needs are related with behavioral and psychological symptoms of dementia (BPSD), caregiver’s age, burden, stress, anxiety and depression [10, 14–16]. However, those results cannot be generalizable to Latin American countries, including Chile, mainly because, as a consequence of the lack of developed health and social services that can meet the needs of persons with dementia, most of the care and cost of the disease relies on informal care [17–19]. Therefore, research on PWD’s needs in non-developed countries can illustrate how met and unmet needs vary according to the provision of health and social services.

The present work is the first study to assess the needs of PWD in a Latin-American country. The aim of this study was to identify met and unmet needs in Chilean older adults with dementia and to determine if there was a relation between their needs and personal, social and caregivers’ factors. It was hypothesized that (a) PWD being looked after by a younger caregiver would have higher number of unmet needs and (b) PWD being cared for by a caregiver with poor mental health (high levels of depressive or anxiety symptoms, and/or burden) would have higher number of unmet needs.

## Method

### Sample

**Fig. 1:**
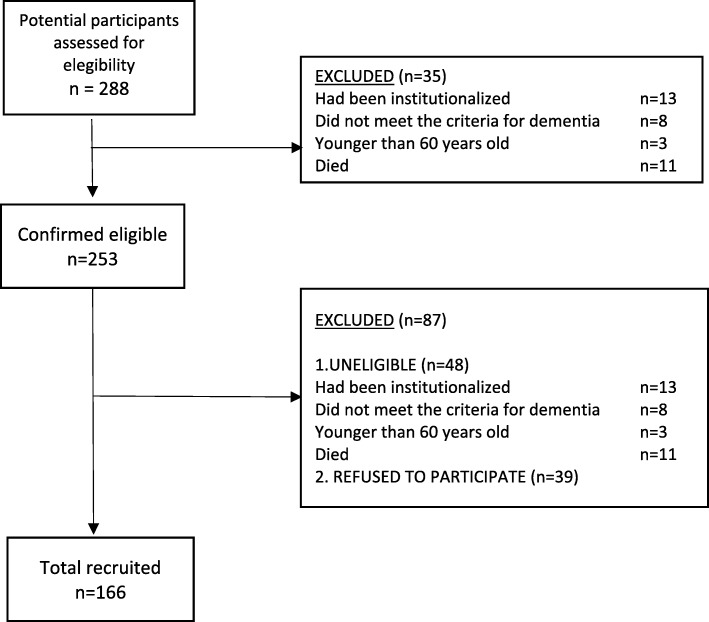
Participants Flowchart

This was a cross-sectional study with a non-probability convenience sampling. One-hundred and sixty-six informal caregivers and their relatives with dementia were interviewed. Initially, 288 potential participants were approached. Out of these, 35 persons were excluded because they did not meet the inclusion criteria as follows: 13 had been institutionalized, 8 did not meet the criteria for dementia, 3 were younger than 60 years old and 11 had died, leaving a total of 253 people suitable to be included in the study. Out of these, 39 could not be reached by researchers and 9 were too confused when contacted by phone that they could not consent to be approached in person. Of the 205 remainder, 39 refused to take part in the study. Some of the reasons for refusing included not wanted to be bothered, not receiving any direct benefit from participating, or reason not known (Fig. 1).

Participants were contacted from different organizations, enrolled through advertisement or referred by a neurologist. The inclusion criteria for informal caregivers were: consider themselves as principal caregivers and not being receiving any formal payment as a caregiver. Criteria for PWD were: being 60 or over, diagnosed with any type of dementia by a physician and being living at their own homes. This research was approved by the Bioethical Committee of the Faculty of Medicine, Universidad de Valparaíso.

### Consent procedure

All interviews were carried out either, at PWD’s or caregiver’s homes. Once there, the interviewer answered any queries and tried to obtain informed consent as follows: a written consent by signing a consent form was required from PWD and their caregivers. Some persons with dementia were unable to provide written informed consent. When this occurred, the interviewer obtained caregiver’s consent and also sought the person with dementia’s assent. During this process, the interviewer made sure that he/she had taken as much time and care in explaining the information about this research as simply as possible. The interviewer avoided using long sentences and attempted to reduce any distractions. To find out if the participants have understood the information given, the interviewer observed their ability to ask any relevant questions and also requested the participant to repeat back the information and how it would relate to them. In addition, the interviewer clarified any doubts about the study and reiterated about the right to withdraw at any time.

### Interviews

Whenever possible, the person with dementia and the caregiver were interviewed separately. Only few caregivers wanted to be present at PWD’s cognitive impairment assessment (11%). Interviews were terminated immediately in presence of any sign of discomfort. Using standardized instruments, PWD were interviewed about their cognitive status. Caregivers were interviewed about the PWD’s sociodemographic details, needs, behavioral and psychological symptoms and functional status. In addition, caregivers were assessed about their own sociodemographic details, depression, anxiety, burden and social support. This interview took about 1 h and a half. The interviewer started the assessment reminding participants what the interview was about and answering any questions they might have. After that, informed consent was sought according to the procedure stated above. There was no particular order to administer the instruments, but usually the interview with the caregiver started with the instrument for assessing needs (CANE).

It is important to mention that the data for this study was collected previous to the recent pilot implementation of national policies regarding PWD’s care.

### Materials

#### Instruments administered to the person with dementia

##### Mini mental state examination (MMSE)

This test assesses cognitive function. It has been widely used in clinical and research practice. The Chilean validation of the MMSE showed a high sensibility 93.6% (95% CI 70.6–99.7%) and specificity 46.1% (95% CI 34.7–57.8%) [20].

#### Instruments administered to the caregiver to obtain information about the person with dementia

##### Camberwell assessment of need for the elderly (CANE)

A comprehensive tool which offers a structured evaluation of needs in older people in 24 areas of social, psychological, physical, and environmental needs rated as no need, met need or unmet need. It assesses the needs of older people from the perspective of the PWD, the caregiver, the staff and the researcher. The CANE has shown good levels of reliability (a = 0.99) and validity (correlated with the CAPE-BRS, *r* = 0.66; and the Barthel *r* = − 0.53) [21]. The Spanish version had good reliability (inter-observer 0.60–1 and test retest 0.65–1). Also it had a criterion validity coefficient of 0.81 [22]. In our study, only caregivers’ report about PWD’s needs have been considered.

##### Barthel index of activities of daily living

This tool assesses functional status of a person. Its scores can be categorized into five levels: total, severe, moderate or mild dependence, and autonomy. The instrument has shown good reliability: intra-observer Kappa indexes between 0,47 and 1, inter-observer concordance Kappa indexes between 0.84 y 0.97 and Cronbach’s alpha coefficient between de 0.86 and 0.92 [23].

##### *Neuropsychiatric inventory questionnaire (NPI-Q)*

The NPI-Q is a structured interview designed to assess a broad range of behavioral and psychological symptoms commonly encountered in PWD. This tool has shown high internal consistency reliability for the frequency/severity product scores (a = 0.88) and for the specific severity (a = 0.87) and frequency (a = 0.88) ratings [24]. The brief version of this scale, the NPI-Q, was validated in Chile with excellent results [25]. The test-retest showed a significant correlation for total symptoms scale (*r* = 0.89) and for distress scale (*r* = 0.90). Convergent validity between NPI-Q and NPI revealed a high correlation for total symptoms and for distress scale (*r* = 0.88; *r* = 0.92) [26].

#### Instruments administered to the caregiver to obtain information about themselves

##### ZARIT burden interview (ZARIT)

This tool measures the stress perceived by caregivers about activities related to the support and care of a relative. It is composed by 22 items and the Chilean validation performed remarkable values of internal consistence (0.87), inter observer reliability (0.86) and test retest reliability (0.91) [27].

##### Hospital anxiety and depression scale (HADS)

This scale was developed to assess anxiety and depression symptoms in no clinical populations. The scale has 14 items divided into two subscales: anxiety (HADS-A) and depression (HADS-D) each one includes 7 items. The Spanish version has a test-retest reliability coefficient over 0.85. Its internal consistency was 0.86 (HADS-A) and 0.86 (HADS-D) [28].

##### Multidimensional scale of perceived social support

This tool measures the characteristics of the social network according to the perception of the subjects. It is composed by 6 items scored from 1 to 4 (1: Never, 2: Sometimes, 3: Frequently, 4: Always). It has shown good psychometric properties with a Cronbach’s alpha from 0.70 to 0.79 [29]. The Chilean validation of this instrument was done with elderly population revealing a good reliability (Cronbach’s 0.86) [30].

### Data analysis

A statistical analysis was performed with IBM SPSS Statistics 21 software package [31, 32]. Descriptive statistics were carried out to assess demographic information and characteristics of met/unmet needs (mean, standard deviation, range and frequency in percentages). The factors associated with unmet needs were analysed using Spearman correlation. Mann-Whitney test was done for the variables caregiver’s sex and PWD’s sex and Kruskall Wallis test was performed with the variable “relationship with the PWD”. All analyses were carried out at significance level of *p* < 0.05 and *p* < 0.01. In addition, a stepwise multiple linear regression analysis was done to determinate the predictors of unmet needs of PWD. The dependent variable was the unmet needs of PWD and independent variables were PWD’s functional status, and caregiver’s social support, burden, age, relationship with PWD, anxiety and depressive symptoms.

## Results

### Demographic and clinical characteristic of people with dementia

Demographic and clinical characteristics of PWD are shown in Table 1. The age of PWD ranged from 63 to 108 years (M = 80.3, s.d. = 7.9). There were 66.7% of women and 33.3% were men. A similar percentage of PWD were either married/with partner or widowed (48.8 and 47.6% respectively). Related to the educational background, 40.2% of older adults with dementia did not complete primary school.

**Table 1:** Demographic and Clinical Characteristic of People with dementia

Characteristic	N		%	95% IC	Mean/s.d. 95% IC
Age	165	60–73	20	13.9–26.7	
		74–83	45.5	38.2–53.3	80.3/7.9
		84–113	30.9	26.7–42.4	(79.04–81.38)
Sex	165	Female	66.7	59.4–73.9	
		Male	33.3	26.1–40.6	
Marital status	164	Single	2.4	0–6.0	
		Married/ Living with a	48.8	38.1–59.5	
		partner	1.2	0.3.6	
		Separate/Divorced	47.6	36.9–57.1	
		Widow			
Cognitive Impairment	141	Severe	18.9	12.4–25.4	14.9/8.04
		Moderate	55.9	47.7–64.1	(13.57–16.22)
		Mild	25.2	18.0–32.4	
BPSD prevalent symptoms	164	Depression/dysphoria	79.9	73.8–86.0	6.6/2.62
		Anxiety	72.6	65.9–79.3	(6.16–6.95)
		Apathy	69.5	62.2–76.2	
Functional Status	163	Total dependence	18.4	12.4–24.3	
		Severe	14.7	9.2–20.1	
		Moderate	12.9	7.7–18.0	57.4/33.01
		Mild	44.7	37.1–52.3	(52.33–62.46)
		Autonomous	9.2	4.8–13.6	

The results of the mini mental (MMSE) showed that 18.9% of them had severe cognitive impairment, 55.9% had moderate and 25.2% had mild cognitive impairment. The results of the Barthel Index showed that 18.4% had total dependence, 14.7% severe dependence, 12.9% moderate dependence, 44.7% mild dependence and 9.2% were autonomous. Finally, the most frequent behavioral and psychological symptoms were depression/dysphoria (79.9%), anxiety (72.6%) and apathy (69.5%).

### Demographic and clinical characteristic of caregivers

Table 2 shows demographic and clinical characteristics of caregivers. The mean age was 57.6 (s.d. = 14.5) and ranged from 22 to 96 years. Most of the participants were women (81.9%) and have a spouse/partner (66.9%). Around 55% were a son/daughter and 28.3% were spouses of the PWD. Regarding the clinical characteristics of caregivers, 68.9% (95% IC = 61.5–75.2%) presented intense burden, 60.7% (95% IC = 53.4%-68–1%) had depressive symptoms and 66.9% (95% IC = 59.5–74.2%) of them had anxiety symptoms.

**Table 2:** Demographic Characteristic of Caregivers

Characteristic	N		%	95% IC	Mean/sd 95% IC
Age	165	21–40	13.9	9.1–19.4	
		41–60	46.6	38.8–53.9	57.58/14.52
		61–80	36.4	29.1–43.6	(55.33–59.97)
		81–100	3	0.6–6.1	
Sex	166	Female	81.9	76.4–88.5	
		Male	18.1	11.5–23.6	
Marital status	166	Single	18.7	12.7–24.7	
		Married/ Living with a partner	66.9	59.6–74.1	
		Separate/Divorced	11.4	6.6–16.9	
		Widow	3	0.6–6.0	
Relationship with PWD	165	Spouse	28.3	21.8–35.8	
		Daughter/son	54.8	47.3–62.4	
		Other relative	15	9.1–20.6	
		Friend	1.8	0–4.2	

### Needs

The mean of total number of needs was 13.3 (s.d. = 4.52; range 0–25) and out of these 10 were met needs (s.d. = 3.86; range 0–20) and 3.3 were unmet needs (s.d. = 2.82; range 0–13). The frequency of CANE met and unmet needs by area are shown in Tables 3 and 4. The most frequent met needs of PWD were found in the environmental area: “Money” (82.5%), “Looking after home” (81.3%), “Food” (78.9%). Some physical needs were also mostly met, such as “Self-care” (75.3%) and “Physical health” (57.2%).

**Table 3:** Frequency (%) of met and unmet needs of PWD (reported by caregivers)

Needs of PWD	N	Met (%)	Unmet (%)	Total (%)
Accommodation	163	28.9	8.44	37.3
Looking after home	163	81.3	2.4	83.7
Food	163	78.9	0,6	79.5
Self-Care	162	75.3,	3.0	78.3
Caring for another	161	9.6	2.4	12.0
Daytime Activities	163	39.8	39.2	79
Memory	161	51.8	34.9	86.7
Eyesight/Hearing	158	42.8	15.7	58.5
Mobility	161	51.8	12.7	64.5
Continence	163	53.6	7.2	60.8
Physical Health	163	57.2	3.64	60.8
Drugs	162	30.1	2.4	32.5
Psychotic Symptoms	162	31.9	16.9	48.8
Psychological Distress	162	39.2	21.1	60.3
Information	162	22.3	27.1	49.4
Deliberate Self-harm	163	9.6	1.8	11.9
Accidental Self-Harm	163	43.4	4.2	47.6
Abuse/Neglect	163	6.6	0.6	7,2
Behavior	163	13.9	3.6	17.5
Alcohol	163	43.6	1.2	4.8
Company	163	24.1	36.1	60.2
Intimate Relationships	163	21.1	3.6	24.7
Money	163	82.5	2.4	84.9
Benefits	162	36.7	13.9	50.6
Mean (s.d.)		10 (3.8)	3.3 (2.8)	13.3 (4.5)

**Table 4:** Caregivers´ factors related with unmet needs of People with Dementia

	Correlation	*p*-value
PWD factors		
Functional status (Barthel)	Rs = −.177	.024*
Caregiver factors		
Age	Rs = −.216	.005**
Relationship with PWD	K-W = 7.307	.026*
Social Support (SS)	Rs = −.383	.001**
Burden (Zarit)	Rs = .372	.001**
Anxiety Symptoms (HADS-A)	Rs = .260	.001**
Depressive Symptoms (HADS-D)	Rs = .354	.001**

Rs = Rho de Spearman. K-W = Kruskal Wallis test. Significant relations *p < .05 **p < 0.01

The most common unmet needs were social and psychological, such as “Daily activities” (39.2%), “Company” (36.1%), and “Memory” (34.9%); followed by “Information” (27.1%) and “Psychological distress” (21.1%).

### Factors associated with unmet needs

Table 4 shows there  was a significant relation between low level of caregivers’ social support and more unmet needs in PWD (r_s_ = −.383; *p* < 0.01). Moreover, caregivers who were children of the PWD (K-W = 7.307; *p* < 0.05) and younger (r_s_ = −.216; *p* < 0.01) reported higher number of unmet needs. Additionally, high levels of caregivers’ burden (r_s_ = .372; *p* < 0.01), depressive symptoms (r_s_ = .354; *p* < 0.01) and anxiety symptoms (r_s_ = .260; *p* = 0.01) were related with higher number of unmet needs in the person with dementia. The only factor of the PWD related with unmet needs was functional status. Higher dependence in the PWD was associated with higher number of unmet needs (r_s_ = .177; *p* < 0.05).

### Predictors of unmet needs

A stepwise multiple linear regression analysis was carried on to determinate which of the factors were the best predictors to unmet needs (Table 5). The dependent variable was the number of unmet needs of PWD (reported by their caregivers). The independent variables were PWD’s functional status, caregivers’ age, social support, burden, anxiety and depressive symptoms and their relationship with PWD. Low social support (*p* < 0.01), high levels of burden (*p* < 0.05), younger caregivers (*p* < 0.05) and caregivers’ anxiety symptoms (*p* < 0.05) were predictors of higher unmet needs of PWD (F = 11.629; *P* < 0.01; *R*^2^ = 23%).

**Table 5:** Predictors of unmet needs (Multiple Linear Regression Model). Dependent Variable: Unmet Needs of people with dementia

Variable	Beta	(p)
Social Support (SS)	−.163	.001
Burden (Zarit)	.031	.030
Caregiver’s age	−.034	.016
Anxiety Symptoms (HADS-A)	.122	.029
Variance explained by model R2%		23%
Adjusted R2%		21%
F=		11.538
Significant (p)		< 0.01
Variables Excluded of the model		Functional status (Barthel)
		Relationship with PWD
		Depressive Symptoms (HADS-D)

Social support and anxiety symptoms were the independent variables with the highest explanation power (β = − 0.163; β = 0.122, respectively). Multicollinearity was no present within the model.

## Discussion

To our knowledge, the present study was the first in Latin America to assess the needs of people with dementia. Met and unmet needs of PWD were identified as well as factors associated with unmet needs.

Regarding the characteristics of PWD, it should be noted that, in our sample, the prevalence of BPSD was higher compared to the one found in community samples from developed countries, however, it is similar to other Chilean study [6]. The high frequency of BPSD could be explained because of the limited services available for people with dementia. In Chile, there is no provision of adequate pharmacological and non-pharmacological interventions aimed at managing BPSD. Since a strong association between BPSD and burden has been found in Chilean caregivers [6], it is important to address this issue through the implementation of the National Dementia Plan.

### Unmet needs of people with dementia in Chile

In line with European samples where caregiver reports on the CANE were used, in our study, the most common PWD’s unmet needs, reported by caregivers, were found in the areas of daytime activities, company, psychological distress and memory [10, 33–36]. This result is consistent with a recent review about PWD’s needs which pointed out that the most frequent unmet needs in this group are the same across countries irrespective of different supply and functioning of services [37]. In addition, in our sample, the mean number of unmet needs was higher than the average shown in most of European studies [10, 35, 36]. This could be reflecting both, a lower provision of services and the fact that most of caregiving assistance relies on family caregivers, consequently resulting in a higher number of unmet needs. Regarding this, it is important to mention that, in order to meet PWD’s and caregiver’s needs, Chilean public policies about dementia should try to address issues that currently are challenges for services in developed countries such as, adequate availability and timing, personalized and flexible offer, appropriate information and coordination, etc. [37].

### Factors associated with unmet needs

The study showed that the most relevant met needs of PWD were related to the environmental and physical areas of dementia. These results are consistent with previous studies in high income countries, which revealed that basic needs of PWD are usually covered (food, home, personal care and physical health) [10, 35, 36]. A possible explanation is that those types of needs are easily identifiable and in most cases can be met by family caregivers without any additional support. In contrast, higher numbers of unmet needs were found in social and psychological areas. Considering caregivers’ own burden and their mental health deterioration, providing PWD with daily activities to perform, information about their condition, help with memory loss and also company and support to psychological distress, could be aspects difficult to address. Usually, approaches to address dementia in Public Healthcare System are focused on older people’s physical health, however, several studies have pointed out the relevance of psychosocial aspects in PWD [8, 38]. The implementation of day-care centres for older adults with dementia is a relevant initiative to acknowledge the lack of activities and social isolation in this group. These centres not only improve the quality of life of PWD, through the development of social and physical activities in a safe environment, but also have a positive impact on caregivers by giving them respite time and consequently decreasing psychological distress [39, 40]. In Chile, this type of centres is recently being piloted in few cities of the country, and its number is expected to grow as long as they are included in the future National Care System.

### Predictors of unmet needs

According to the results of this study, unmet needs of PWD were predicted only by caregivers’ factors. Our two hypotheses were confirmed. Thus, caregivers’ higher level of anxiety symptoms and burden, low perceived social support and younger age were related to a higher number of unmet needs in people with dementia. Different studies of Chilean caregivers of PWD have shown they have intense burden and high levels of depressive and anxiety symptoms [6, 41]. It is not surprising that caregivers with these mental health characteristics were not in good condition for both, performing caregiving tasks and addressing unmet needs of those who they look after to, particularly in the psychosocial area. In Chile, most older people expect being cared for by their families [42]. In addition, worldwide, it has been encouraged that, in order to have better health outcomes, older adults should stay at home for as long as possible [35]. However, this can be achieved only under the presence of a health and social care system which provides strong support for PWD and their caregivers. In Chile, a relevant initiative targeting this matter is being piloted, a new public programme called “Chile Cuida” (“Chile Provides Care”). This programme intends to give some relief to informal caregivers, to create new jobs and to promote better conditions for older people to stay in their own households. People who live within the family caregiver’s neighborhood or community are trained to become a formal caregiver, making them capable and formally prepared to provide assistance to PWD in their households twice per week for 3 or 4 h a day. This visit gives respite to family caregivers who also have the opportunity to participate in workshops with health professionals, training sessions or recreational activities [34]. In addition, the National Dementia Plan has been recently launched by the Minister of Health and is starting to be piloted in few cities of the country. This policy aims to address psychosocial aspects of dementia, for example, through the implementation of day-care centres across the country, improving the coordination between primary and secondary health and giving treatment to the PWD-caregiver dyad. [9].

Caregiver’s age was also found to be a predictor of PWD’s unmet needs. Younger caregivers are usually daughters of PWD who already have multiple responsibilities. It has been found that younger caregivers usually have to take care of their own children, comply with professional duties and social life events. Additionally, they tend to experience less feelings of reward from being a caregiver and show higher levels of burden compared to older caregivers [43]. These issues add difficulties to meet PWD’s unmet needs.

#### Limitations and strengths

To our knowledge, the present study is the first to assess the needs of older PWD in a Latin American country (Chile). It was carried out with a relevant sample size and included measures for community-dwelling PWD and caregivers. Nevertheless, there are some limitations and suggestions to consider for further research. Even though all the participants had a degree of cognitive impairment, dementia diagnosis was not always given by a specialist physician (geriatrician, neurologist, etc) which might have biased the selection of participants [44]. Despite this, the sample is representative of Chilean reality where most PWD get their first approach to a diagnosis by a general practitioner, not by a specialist. In addition, this research considered older people with any kind of dementia. It is suggested that future research focuses on persons with specific types of dementia whose needs could differ according to particular diagnoses [45, 46]. We should be cautious to extrapolate our results to all dementia patients since our sample comprises subjects who do have a diagnosis. Other studies show that roughly one half of those, who would meet the diagnostic criteria for dementias, have been diagnosed [47, 48]. According to current evidence, patients with a diagnosis of dementia are more severely impaired therefore could present more needs compared to under-diagnosed subjects [49]. Also, the sample of our study was a convenience sample, known to services and from urban areas, consequently, the results cannot be generalized to Chilean PWD not known to services and/or living at rural areas. In order to reflect the prevalence of needs, future research could consider an epidemiological population-based sample therefore making the results more generalizable.

Finally, in this study, PWD’s needs where rated by caregivers and thus, their views on the needs of their relative could have been influenced by their own physical and mental health. However, literature has shown that, even though PWD rate less unmet needs than their caregivers, they agree on most frequent areas where they are not receiving the right help [10, 35]. Future research could include a description of needs according to the point of view of older persons with dementia. In addition, it is important to mention that caregiver’s factors and caregiver’s perspective on PWD’s unmet needs could be mutually affected. For example, a high level of caregiver’s burden could lead to a higher number of PWD’s unmet needs or viceversa. Previous studies using the CANE have considered, indistinctly, the concept of “unmet needs” either, as a result or as a predictor of caregiver’s state [10, 33, 36, 50]. Longitudinal research is needed in order to determine causality.

## Conclusion

The results of this research revealed the urgent need to address the psychological and social aspects of dementia, because most frequent PWD’s unmet needs and their predictors pointed out to deficiencies in those areas. The fact that only caregivers’ factors were predictors of PWD’s unmet needs confirms the relevance of treating both, the PWD and their caregivers, as a dyad who influences each other. Caregiver’s lack of social support, high levels of burden, presence of anxiety symptoms and lower age were predictors of unmet needs in people with dementia. Considering these results, it is expected that, in the years coming, recently implemented national policies and care initiatives, could meet most common PWD’s unmet needs both, directly and through addressing caregivers’ issues. In order to achieve this, it is very important, first, to expand and evaluate the implementation of those policies, and second, carrying out future research on needs to find out whether they are being addressed or not.

## Abbreviations

BPSD: Behavioral and psychological symptoms of dementia
CANE: Camberwell Assessment of Need for the Elderly
HADS: Hospital anxiety and depression scale
MMSE: Mini mental state examination
NPI: Neuropsychiatric Inventory
PWD: People/person with dementia
Rs: Spearman correlation
S.D.: Standard deviation
ZARIT: Zarit Burden Interview
